# Oxycodone Self-Administration Induces Alterations in Expression of Integrin, Semaphorin and Ephrin Genes in the Mouse Striatum

**DOI:** 10.3389/fpsyt.2018.00257

**Published:** 2018-06-12

**Authors:** Vadim Yuferov, Yong Zhang, Yupu Liang, Connie Zhao, Matthew Randesi, Mary J. Kreek

**Affiliations:** ^1^Laboratory of the Biology of Addictive Diseases, The Rockefeller University, New York, NY, United States; ^2^Research Bioinformatics, Clinical and Translational Science Award, The Rockefeller University, New York, NY, United States; ^3^Genomic Resource Center, The Rockefeller University, New York, NY, United States

**Keywords:** oxycodone, RNA seq, axon guidance genes, cell type enrichment, integrins

## Abstract

Oxycodone is one a commonly used medication for pain, and is also a widely abused prescription opioid, like other short-acting MOPr agonists. Neurochemical and structural adaptations in brain following chronic MOPr-agonist administration are thought to underlie pathogenesis and persistence of opiate addiction. Many axon guidance molecules, such as integrins, semaphorins, and ephrins may contribute to oxycodone-induced neuroadaptations through alterations in axon-target connections and synaptogenesis, that may be implicated in the behaviors associated with opiate addiction. However, little is known about this important area. The aim of this study is to investigate alterations in expression of selected integrin, semaphorin, ephrins, netrin, and slit genes in the nucleus accumbens (NAc) and caudate putamen (CPu) of mice following extended 14-day oxycodone self-administration (SA), using RNAseq.

**Methods:** Total RNA from the NAc and CPu were isolated from adult male C57BL/6J mice within 1 h after the last session of oxycodone in a 14-day self-administration paradigm (4h/day, 0.25 mg/kg/infusion, FR1) or from yoked saline controls. Gene expressions were examined using RNA sequencing (RNA-Seq) technology. RNA-Seq libraries were prepared using Illumina's TruSeq® Stranded Total RNA LT kit. The reads were aligned to the mouse reference genome (version mm10) using STAR. DESeq2 was applied to the counts of protein coding genes to estimate the fold change between the treatment groups. False Discovery Rate (FDR) *q* < 0.1 were used to select genes that have a significant expression change. For selection of a subset of genes related to axon guidance pathway, REACTOME was used.

**Results:** Among 38 known genes of the integrin, semaphorin, and ephrin gene families, RNA-seq data revealed up-regulation of six genes in the NAc: heterodimer receptor, integrins *Itgal, Itgb2*, and *Itgam*, and its ligand semaphorin *Sema7a*, two semaphorin receptors, plexins *Plxnd1* and *Plxdc1*. There was down-regulation of eight genes in this region: two integrin genes *Itga3* and *Itgb8*, semaphorins *Sema3c, Sema4g, Sema6a, Sema6d*, semaphorin receptor neuropilin *Nrp2*, and ephrin receptor *Epha3*. In the CPu, there were five differentially expressed axon guidance genes: up-regulation of three integrin genes, *Itgal, Itgb2, Itga1*, and down-regulation of *Itga9* and ephrin *Efna3* were thus observed. No significant alterations in expression of Netrin-1 or Slit were observed.

**Conclusion:** We provide evidence for alterations in the expression of selective axon guidance genes in adult mouse brain following chronic self-administration of oxycodone. Further examination of oxycodone-induced changes in the expression of these specific axon guidance molecules and integrin genes in relation to behavior may provide new insights into development of addiction to oxycodone.

## Introduction

Oxycodone is one of the most commonly use medications for pain, and like many short-acting MOPr agonists, it also has abuse potential. The neuroadaptations in specific brain regions following chronic opioid administration occur at the neurochemical and structural levels, and may underlie opioid use disorders. Like other drugs of abuse, opioids have the ability to cause neuroplasticity by altering morphology of dendrites and spines, which are the primary sites of excitatory synapses in brain regions involved in incentive motivation, reward, and learning (1). A decrease in the complexity of dendritic branching and number of dendritic spines on neurons specifically located in the nucleus accumbens (NAc) and cortex of rats was found following morphine self-administration (2, 3). Molecular mechanisms that underlie drug-induced structural alterations are still not fully understood. However, there are pharmacological and genetics evidence that the axon guidance genes may contribute to these morphological alterations (4, 5).

Researchers have identified five families of canonical guidance proteins: semaphorins, ephrins, slits, netrins, and integrins (4, 6). Proteins of the axon guidance gene family may contribute to oxycodone-induced neuroadaptations, through alterations in axon-target connections and synaptogenesis, and these may be implicated in the behaviors associated with opioid use disorders. Originally, integrins, semaphorins, ephrins, slits, and netrins with their cognate receptors were found to be implicated in establishing functional neural circuits, cell migration, and synapse formation during development (5). However, axon guidance molecules are also expressed in adult brain, and may contribute to alterations in neural circuit regulation, throughout axon pruning, synaptogenesis, dendrite, and spine morphogenesis. Several studies showed an implication of axon guidance proteins in neuroadaptation following drugs of abuse administration, and response to brain injury (7–12).

EPH receptor tyrosine kinases are divided into two classes, EPHA receptors (A1–A8, A10) and EPHB receptors (B1–B4, B6), based on their binding affinities for ligands ephrin-A (A1–A5) or ephrin-B (B1–B3) ligands (13). Eph receptors and ephrins mediate bidirectional signaling, being both membrane proteins. In general, cellular response to the Eph receptor activation is local actin fiber depolymerization, which results in rapid cytoskeletal collapse and loss of focal adhesions, leading to cell detachment. EphB1 receptor and ligand ephrin-B2 ligand are expressed in the midbrain dopaminergic neurons. Activation of the EphB1 inhibits the growth of neurites and induces the cell loss of substantia nigra, but not ventral tegmental, dopaminergic neurons (14). The same study showed that ephrin-B2 expression is upregulated by cocaine or amphetamine in the striatum of adult mice, suggesting that ephrin-B2/EphB1 interaction may play a role in drug-induced plasticity. Of interest, Liu et al showed that escalating morphine treatment up-regulates expression of EphB1 in the mouse spinal cord, and that EphB2 blocker (EphB2-Fc) attenuated most of naloxone-precipitated morphine withdrawal signs (15).

Twenty semaphorins fall into five classes, semaphorins 3–7 (16). Class 3 semaphorins are secreted proteins, classes 4–6 are transmembrane proteins, and semaphorin 7A is linked to the plasma membrane via a glyco-phosphatidylinositol (GPI) anchor. Most of the effects of semaphorins are mediated by plexins, a group of nine transmembrane receptors that can be subdivided into four classes, plexins A–D, and two neuropilin receptors, Nrp1 and Nrp2. The semaphorin–plexin system is involved in multiple functions during development and in the adult organism, particularly in the nervous system, the immune system and during angiogenesis. Deregulation of semaphorin expression was documented in many pathological conditions such as ischemia, degenerative diseases, multiple sclerosis (5, 16). Semaphorins have also been shown to have immune regulatory functions in B and T cells. *In vitro*, Sema4d enhanced B cell survival, and play a role in monocytes and macrophages migration. Recently, Sema7A was identified as an effector molecule in T-cell-mediated inflammation through an integrin-mediated mechanism (17).

Integrins are a large family of receptors for components of the extracellular matrix (ECM). Integrins are obligatory αβ-heterodimers that undergo large conformational changes in their extracellular domains in response to signaling events inside cells. In mammals, there are 18 different α-subunits and 8 different β-subunits, which together generate 24 distinct integrin αβ-heterodimer receptor combinations (18). Intracellularly, integrins link to the actin cytoskeleton via adaptor proteins, such as talin and vinculin, and engage second messenger signaling cascades through several kinases such as Srk, ILK, FAK, and PI3K. Given the diversity of integrin effects, regional differences in receptor expression could be involved in activity-dependent synaptic plasticity, including activity-dependent neural circuit adaptation, and in turn its dysfunctions might promote neurological disorders (19). In relation to drug addiction-like behavior, inhibition of integrin-linked kinase (ILK) in the rat NAc core blocked the induction of cocaine psychomotor sensitization, and prevented cocaine-induced increase in dendritic density and dendritic spine numbers (20). It has been shown that the basal levels of the integrin beta-1 (Itgb1) were elevated after chronic cocaine administration (11). Alpha and beta integrins are receptors for semaphorin 7A, and mediate its function (4). This supports potential involvement of integrins in the chronic cocaine-induced behaviors. However, there are no reports regarding an involvement of integrins in the opioid-induced pathology in humans or animal models.

Netrins play various important roles in the correct wiring of the nervous system during development (21). To date, several netrins have been described: netrin-1, netrin-3, netrin-4 and G-netrins. The most studied netrin-1 induces axon outgrowth via one of its receptors DCC (Deleted in Colorectal Cancer) in several types of neurons. Recent morphological analyses suggested a possibility of a shift in the function of netrin-1 in cortical axons during development, from promotion of outgrowth to promotion of branch formation (22). Function-blocking experiments suggested that DCC may contribute not only to axon outgrowth but branching. There is no information on modulation of opioid-induced behaviors by netrin-1 or its receptor DCC expression. Of interest, *in vitro* treatment of neurons of dorsal root ganglion (DRG) with netrin-1 stimulates translation of the kappa opioid receptor (KOR), which activates its downstream target the focal adhesion kinase (FAK) (23).

Another class of axon guidance proteins is Robo receptor and its ligand Slit. The Slit**/**Robo pair not only functions in axon guidance in development but also in diverse processes in the CNS, like cell migration, axonal branching, axonal targeting or cell differentiation (24). In most vertebrates there are 3 Robo receptors expressed in CNS cells, Robo-1, Robo-2, and Robo-3. Three Slit genes have been identified in mammals, Slit 1-Slit-3. Full-length Slits can be cleaved by proteases generating shorter functional N-terminal isoforms (Slit1-N, Slit2-N, and Slit3-N). In relation to drug of abuse area, several studies showed their involvement in regulation of dopaminergic neurons (25, 26). For example, Slit-2 inhibits growth of tyrosine-ir positive (TH+) axons in primary cultures of the rat ventral midbrain. Similarly, Slit-2 reduced the number and length of TH+ axons in explants from the ventral midbrain tissue of mouse brain (26). However, little is known about the effect of MOPr agonist self-administration on these important guidance molecules (10).

Nonmedical use and misuse of prescription opioids, including oxycodone is an increasing public health problem (27, 28). We hypothesize that specific representatives of the axon guidance gene family are implicated in development of neurobiological adaptation that occurs during chronic oxycodone self-administration. The aim of this study is to identify alterations in expression of specific axon guidance genes in the nucleus accumbens and caudate putamen of mice, following chronic oxycodone self-administration using RNA-seq technology.

## Methods

### Animals and oxycodone self-administration procedure

Male adult (11 weeks old) C57BL/6J mice were obtained from Jackson Laboratory, Bar Harbor, ME. Animal care and experimental procedures were conducted according to the Guide for the Care and Use of laboratory Animals (Institute of Laboratory Animal Resources Commission on Life Sciences 2016). Animals had free access to food and water in a light (12:12 h) reverse cycle, lights on at 7:00 p.m. and off at 7:00 a.m. Mice were handled prior to surgery. Catheter implantation for drug self-administration was carried out after acclimation of animals for 7 days. The experimental protocol used was approved by the Institutional Animal Care and Use Committee of the Rockefeller University.

### Oxycodone self-administration

Details of surgery and catheter implantation, and subsequent the procedure of oxycodone self-administration (SA) in mice has been described previously (29). Briefly, the self-administration experiments were carried out in chamber ENV-307W (21.6, 17.8, and 12.7 cm; Med Associates, St Albans, VT). A 4-h self-administration session was conducted once a day for 14 consecutive days with oxycodone, *n* = 6 (0.25 mg/kg/infusion) or yoked saline controls (*n* = 6).

### RNA extraction

Mice were sacrificed within 1 h after the last session of oxycodone self-administration, by exposure to CO_2_. The brain tissues of 12 mice (6 oxycodone and 6 yoked saline controls) were used for the RNA-seq study. Total RNA was isolated from the nucleus accumbens (NAc) and caudate putamen (CPu) using the miRNeasy Kit (Qiagen, Valencia, CA). Agilent 6000 RNA Nano Chips were used to examine the integrity of RNA in samples.

### RNA-seq library preparation and sequencing

RNA-seq library preparation and sequencing of samples isolated from the CPu was performed by LC Sciences (Houston, TX), whereas RNA-seq library preparation and sequencing of RNA isolated from NAc was performed by the Genomic Resource Center at the Rockefeller University. Both RNA-seq libraries were prepared using Illumina's TruSeq® Stranded Total RNA LT kit following the manufacturer's protocol. Libraries were validated using Agilent Tape Station High-Sensitivity RNA kits and normalized. Libraries were multiplexed, four samples per lane, and sequenced. An Illumina HiSeq 2500 apparatus was used to obtain 100 bp single end reads for samples from the nucleus accumbens, whereas Illumina HiSeq 2000 was used to obtain 100 bp paired-end reads for samples from the CPu. All samples were analyzed exactly as described previously (30). Briefly, the reads were aligned to the mouse reference genome (version mm10) using STAR (31) aligner with default parameters. The alignment results were evaluated through RNA-SeQC (32). Aligned reads were then summarized through featureCounts (33) with the gene model from Ensemble (Mus_musculus GRCm38.75.gtf) at gene level.

Samples were normalized through a set of housekeeping genes *Ppia, Gusb*, and *Gapdh* as described previously (30). Principal Component Analysis (PCA) was then applied to normalized counts of all the samples by brain region, to detect outlier samples. In the CPu, data from one saline control, and from one oxycodone-treated animals were thus excluded from downstream analysis. In the NAc, data from one oxycodone-treated animal was excluded from the downstream analysis.

### Axon guidance gene selection

REACTOME (linked to KEGG) was used to identify 416 genes in the NAc and 445 genes in CPu in the axon guidance canonical pathway (stable Identifier R-MMU-422475.1). Statistical significance and fold change of oxycodone-induced alterations in expression of 32 axon guidance-related genes (integrins, semaphorins and ephrins, and their receptors) were extracted from the total list of differentially expressed genes in the RNA-seq data for the NAc and CPu in this study (Supplement Tables 1s, 2s, respectively).

### Analyses of cell-type specific enrichment of integrin, semaphorin and ephrin gene transcripts

To examine whether DE axon guidance genes may produce their effect in cell type specific manner, we have studied potential enrichment in transcripts of the axon guidance genes in specific cell types such as astrocytes, neurons, microglial and endothelial cells. Publicly available RNA-Seq transcriptome data (34) were downloaded from GEO (GSE52564). For each gene of integrin, semaphorin and ephrin gene families, that had a significant change (FDR < 0.1) in either NAc or CPu, expression fold change of each cell type was calculated as described recently (34) (Table 2). For example, in astrocytes: FC = gene X's expression in astrocytes/average gene X's expression in all non-astrocyte cell types.

## Statistics

### RNA-seq differential gene expression analysis

We used **DESeq2** (https://doi.org/10.1186/s13059-014-0550-8) (35), a method for differential analysis of RNA-seq data to estimate fold-change and significance testing. Specifically, DESeq2 estimate gene-wise log fold of changes (LFCs) between conditions from the standard Generalized Linear Model (GLM) fits to obtain maximum-likelihood estimates, and then fit a zero-centered normal distribution to the observed distribution of Maximum-likehood Estimate (MLE) over all genes. This distribution is used as a prior on LFC in a second round of GLM fits, and the maximum a posteriory (MAP) estimates are kept as final estimates of LFC. For significance testing, DESeq2 uses a Wald test: the shrunken estimate of LFC is divided by its standard error, resulting in a z-statistic, which is compared to a standard normal distribution. The Wald test P values from the subset of genes that pass an independent filtering step, are adjusted for multiple testing using the procedure of Benjamini and Hochberg (36).

DESeq2 was applied to the normalized counts to estimate the fold-change between the samples from mice that had self-administered oxycodone versus those from yoked saline controls, using negative binomial distribution. An adjusted *p*-value of less than 0.1 (FDR < 0.1) and fold change ≥15% were used to select genes that have a significant expression change.

## Results

### Extended access oxycodone self-administration

Oxycodone self-administration behavior in the animals in this sample was reported in our previous publication (30). Our previous studies of oxycodone dose-response effects showed that a dose of 0.25 mg//kg per infusion lead to escalation of oxycodone over the sessions (29). Animals showed a robust escalation of daily oxycodone intake across 14 consecutive daily sessions. A two-way ANOVA for drug condition (oxycodone or saline) × session (days 1–14), showed a significant main effect of drug condition [*F*_(1, 252)_ = 672.6, *p* < 0.0001] and a significant main effect of session [*F*_(13, 252)_ = 2.359, *p* < 0.01]. The average amount of oxycodone self-administered for each mouse increased from 3 mg/kg on the 1st session to 7.5 mg/kg on the last sessions. Oxycodone self-administering mice showed a much greater frequency of response on the active versus inactive holes. In contrast, yoked-saline control mice showed lower responding, and stayed stable across 14 daily sessions.

### Oxycodone-induced alterations in expression of integrin, semaphorin and ephrin genes

#### Nucleus accumbens (NAc)

Among 38 integrin, semaphorin and ephrin genes selected for further analyses, we found significant alterations in 14 genes in the NAc at fold change ≥15%, with a false discovery rate (FDR) of < 0.1, of which six were up-regulated and 8 were down-regulated (Table 1A). Among up-regulated genes, there were integrin receptors *Itgal* and *Itgb2*, and their ligand semaphorin *Sema7a*. There was also up-regulation of semaphorin receptors *Plxdc1* and *Plxnd****1***, with concomitant lower levels in expression of their cognate ligands *Sema3c, Sema4g, Sem6a*, and *Sema6d*. In contrast to elevated expression of the receptor *Plxd1*, the receptor neuropilin *Nrp2* was down-regulated. Two integrin genes, *Itga3* and *Itgb8* had lower level expression in the oxycodone treatment group, compared to the saline group, and only one gene from the ephrin receptor family, *Epha3* was down-regulated in this group.

**Table 1:** Axon gudance and integrin genes with differential expression in the mouse nucleus accumbens and caudate putamen following 14 days of oxycodone self-administration (FDR < 0.1, FC > 15%).

**Gene symbol**	**Fold change**	**Direction**	***p*-value**	**FDR**	**Gene name**
**A. NUCLEUS ACCUBBENS**					
*Itgal*	2.54	↑	0.00000	0.0000	Integrin alpha L
*Itgb2*	1.69	↑	0.00006	0.0068	Integrin beta 2
*Sema7a*	1.40	↑	0.00637	0.0910	Semaphorin 7a
*Plxdc1*	1.32	↑	0.00102	0.0464	Plexin dc1
*Plxnd1*	1.48	↑	0.00376	0.0693	Plexin d1
*Itgam*	1.29	↑	0.00519	0.0852	Integrin alpha M
*Epha3*	0.77	↓	0.00298	0.0620	Ephrin receptor a3
*Sema3c*	0.77	↓	0.00223	0.0618	Semaphorin 3c
*Sema4g*	0.75	↓	0.00482	0.0821	Semaphorin 4g
*Sema6a*	0.82	↓	0.00782	0.1050	Semaphorin 6a
*Sema6d*	0.84	↓	0.06142	0.0583	Semaphorin 6d
*Nrp2*	0.61	↓	0.00165	0.0583	Neuropilin 2
*Itga3*	0.77	↓	0.00244	0.0620	Integrin a3
*Itgb8*	0.86	↓	0.00583	0.0861	Integrin b8
**Gene symbol**	**Fold change**	**Direction**	***p*****-value**	**FDR**	**Entrez gene name**
**B. CAUDATE PUTAMEN**					
*Itgal*	2.53	↑	0.00000	0.0002	Integrin alpha L
*Itgb2*	2.14	↑	0.00003	0.0041	Integrin beta 2
*Itga1*	1.48	↑	0.00060	0.0323	Integrin alpha 1
*Itga9*	0.72	↓	0.00004	0.0042	Integrin alpha 9
*Efna3*	0.71	↓	0.00185	0.0747	Ephrin A3

#### Caudate-putamen (CPU)

As observed above in the NAc, in this region there was strong up-regulation of three integrin genes, *Itgal, Itgb2*, and *Itga1* (Table 1B). Among down-regulated genes, there were integrin *Itga9* and the ephrin receptor ligand, *Efna3*.

#### Cell type specific enrichment analysis of integrin, semaphorin, and ephrin gene transcripts

Recently, an RNA-sequencing transcriptome and splicing database has been reported for purified representative populations of neurons, astrocytes, oligodendrocyte precursor cells, newly formed oligodendrocytes, myelinating oligodendrocytes, microglia, and endothelial cells from mouse cerebral cortex (34). This database provided a platform for analyzing and comparing transcription profiles for various cell classes in the brain herein. We have used this publicly available database to perform analyses of enrichment in expression levels of axon guidance genes that would be expected in astrocytes, neurons, microglial and endothelial cells. The analyses have revealed an enrichment of integrins *Itgal, Itgb2, Itgam*, and receptors *Plxdc1* and *Plxdc2* expression in microglia cells (Table 2). Similar analysis showed that ephrin *Efna3*, and its receptor *Epha3*, and semaphorins *Sema3c* and *Sema4g* are abundoned in neurons. Expression levels of semaphorins *Sema7a* and *Sema3c*, semaphorin receptor *Nrp2*, and integrin *Itga1* were enriched in endothelial cells. Astrocytes were enriched with semaphorin *Sema6d*.

**Table 2:** Cell type enrichment of integrin, semaphorin, and ephrin.

**Transcripts (log2 of Fold Enrichment)**				
**Gene**	**Astrocytes (A)**	**Neurons (N)**	**Microglia (MGL)**	**Endothelia (Endo)**
Itgal	−2.235	−2.235	**2.956**^a^	−1.419
Itga1	−2.179	−4.461	−5.737	**6.439**
Itga3	−2.095	**2.048**	−4.006	**1.321**
Itga9	−5.707	−3.4	**2.081**	−2.568
Itgal.1	−2.235	−2.235	**2.956**	−1.419
Itgam	−8.218	−7.842	**5.905**	−8.752
Itgb2	−7.874	−6.023	**6.834**	−5.103
Itgb2.1	−7.874	−6.023	**6.834**	−5.103
Itgb8	**0.978**	−1.988	−6.774	−6.7
Nrp2	−2.55	0.197	0.802	**1.935**
Plxdc1	−4.265	0.264	**2.822**	0.494
Plxdc2	−1.234	−1.446	**3.347**	−2.651
Plxnd1	−3.509	1.016	−0.667	**3.113**
Sema3c	−3.559	**1.725**	−5.425	**3.192**
Sema4g	−0.77	**2.445**	−0.153	−4.345
Sema6a	**0.642**	−0.977	−7.241	−0.245
Sema6d	**2.287**	−1.622	−3.912	0.376
Sema7a	−3.782	−2.209	−3.772	**4.542**
Efna3	−2.091	**4.598**	−2.668	−2.987
Epha3	−2.207	**4.406**	−3.799	−3.525

a *Example of calculation of a fold enrichment of a gene in a specific cell type: Log2 [expression of X gene in astroglia divided by average expression of X gene in non-astrocyte cell types (N+MGL+Endo)]. Bolded numbers show the highest enrichment of X gene transcripts in a specific cell type. Positive numbers indicate a higher abundance of gene X expression in a particularly cell type, compared with its expression in other cells*.

## Discussion

In this study, we have for the first time RNA-seq technology applied to examine differential gene expression of the axon guidance gene family and integrins, in the NAc and CPu of adult male mice, following 14-day extended-access self-administration of oxycodone, compared with the yoked saline controls. This self-administration regimen resulted in substantial daily intake and escalation of oxycodone across sessions (37).

We have found substantial difference in number of the differentially expressed axon guidance genes in response to the chronic oxycodone self-administration, 14 genes in the NAc, and five genes in the CPu. This difference between these two regions could be due to, in part, difference in neuroanatomical structure and functions of ventral and dorsal striatum in the development of dependence to drugs of abuse, with the NAc implemented in drug rewards, and CPu in drug-induced habituation behavior (38). Earlier studies showed that in contrast to NAc, injections of morphine to CPu did not produce a condition place preference (39). This could be also a result of intrinsic differences in the dopamine fiber responsiveness that innervate these specific regions of the striatum as well as in dopamine transporters in regulation of drug-induced dopamine release. For example, repeated morphine administration at a dose of 1.0 mg/kg (s.c.) increased synaptic dopamine concentrations preferentially in the rat NAc, not in CPu (40, 41). After 15 days of withdrawal after 3 days of morphine treatment, challenge with 1 mg/kg (s.c.) morphine failed to significantly modify extracellular DA in the CPu of sensitized as well as in control rats (41).

We have found oxycodone-induced alterations in expression mainly in three axon guidance gene families such as integrins, semaphorins, and ephrins. These systems may contribute to oxycodone-induced neuroadaptations through alterations in axon-target connections and synaptogenesis and may be implicated in the behavioral and neurobiological adaptations occurring in opioid use disorders. No significant oxycodone-induced alterations in expression of Netrin-1 or Slit were observed. We found here that integrins Itgal and Itgb2 have the greatest increase in expression in both the NAc and CPu immediately after chronic oxycodone SA. However, oxycodone-induced increase of their potential ligand semaphorin Sema7a was observed only in the NAc, but not in the CPu. In the adult brain, many integrins are present at high levels at synapses. The sequence arginine-glycine-aspartic acid RGD (42) was identified as a general integrin-binding motif. Application of soluble GRGDS (Gly-Arg-Gly-Asp-Ser) peptides completely abolished the mu opioid receptor agonist DAMGO inhibitory effect on cyclic AMP (cAMP) accumulation in bradykinin-primed trigeminal ganglia neurons (43, 44). This suggests that activation of specific integrins at focal adhesions may modulate the mu opioid receptor signaling by altering interactions with G proteins. Also, RGD peptides, or anti-integrin antibodies block N-methyl-D-aspartate (NMDA)-mediated excitatory postsynaptic currents in hippocampal neurons (45), suggesting that RGD-binding integrins are important in neurotransmission.

We have found only one gene from the ephrin receptor family, Epha3, which was down-regulated in the NAc of oxycodone-treated mice. In other studies, similar down regulation in the expression of ephrin genes in the rat NAc was observed at 24 h after secession of heroin self-administration (6 h/day for 5 days) (12). In contrast, cocaine treatment increased expression of many ephrin and ephrin receptor in the rat and mouse striatum and hippocampus (14, 7) and in striatum of nonhuman primates (46). Alterations in expression of the Eph/ephrin genes have also been linked to neuropathology ranging from inhibition of neural repair after traumatic injury and stroke to neurodegenerative diseases (47, 48). Ephrin receptors and their ligands are implicated in dendritic spine morphology throughout an interaction with integrins. For instance, activation of the receptor EphA4 by ephrinA3 inhibits activity of integrin Itgb1 and downstream signaling, and leads to decreases spine length and density (49). Transgenic overexpression of ephrinA3 in astrocytes reduces glutamate transporter levels and elevates extracellular glutamate concentrations (50). In contrast, loss of ephrin-A3 raises glutamate transporter currents in astrocytes. Functionally, EphA4 and ephrinA3 modulate transporter glutamate currents in astrocytes.

Previously, microarray studies of morphine-treated mice showed alterations in expression of genes related to the semaphorin pathway in the NAc such as Sema3f, Sema4b, sema6c, Sema6d, Sema7a, and Plxna3 (10) and Sema6a (44). Heroin self-administration induced downregulation of Sema5a, Sema6c and receptor Plxnb1 in the rat NAc (12). Consistent with these earlier reports, in this study we have found oxycodone-induced downregulation of Sema3c, Sema4g, Sema6a, Sema6d, and upregulation Sema7a in the mouse NAc. Our results are also consistent with alterations in the expression of semaphorins and their receptors in post-mortem brain of patients chronically exposed to alcohol or cocaine (51), particularly upregulation of *SEMA7A* and plexin *PLXDC1*, and down-regulation *SEMA4B* in hippocampus. Alterations in expression of semaphorins and their receptors were documented in many pathological conditions such as ischemia, degenerative diseases, multiple sclerosis (5).

Early studies showed that chronic morphine modulate both adaptive and innate immune systems, as well as activate neuroinflammation (52). Several studies showed that this effect of morphine on immune system is mediated by the central MOPr, and can be antagonized by naltrexone (53, 54). Several studies showed that microglia, astrocytes, oligodendrocytes, and endothelial cells actively respond to opioids by producing an inflammatory immune response (55, 56). Recently, we have reported mRNA levels of numerous genes related to the inflammation and immune functions changed as a result of oxycodone self-administration, in the CPu and NAc (30). We have found oxycodone-induced upregulation in the NAc of many glial- and immune cell-specific genes, such as the chemokine receptor Ccr5, chemokine Ccl 12, toll-like receptor Tlr7, interleukin Il1b, interleukin-17 receptor, antigen CD14, antigen CD163, complement component 1 C1qc, interferon regulatory factor Irf1, and others. Astrocytes and microglia release several neuroactive molecules, such as glutamate, D-serine, ATP, GABA, TNFa, that can actively regulate many aspects of neuronal function, including neurotransmitter release, gene regulation, dendritic morphology, and synaptic connectivity (57).

Semaphorins were shown to be involved in initiation of the immune response in brain (58), and alterations in their expression may be related to the oxycodone-induced changes in inflammation/immune gene expression found in this study. Sema7A has been identified as an effector molecule in T-cell-mediated inflammation through an integrin-mediated mechanism, reviewed in (17). Of interest, many immune proteins have been found in healthy, uninfected nervous system, and they may participate in regulation of neuronal functions, including neurotransmitter release, dendritic morphology, and synaptic transmission (59–61).

Several studies proposed axon guidance molecules for contact interaction of neuronal and glial cells (13). The oxycodone-induced differential expression of integrins, semaphorins, and ephrins in ventral and dorsal regions of the mouse striatum implies that they likely participate in the altered communication occurring between neurons and glial cells. Therefore, an identification of cell type specificity of axon guidance gene expression would help in understanding of their specific roles in drug-induced alterations in neuronal activity. We did not perform a cell sorting procedure of brain tissues in our experiments. However, our bioinformatics analysis of oxycodone-induced differentially expressed axon guidance genes showed that their mRNA enrichment varied among neuronal, astrocyte, microglial and endothelial cells, supporting their pleiotropic functions in adaptation to chronic oxycodone treatment.

Since gene expression changes were examined immediately after 14 consecutive days of chronic oxycodone self-administration, it is also not clear whether the changes in axon guidance gene expression are long-lasting, which may play an important role in drug-induced adaptation. The exact molecular mechanisms of regulation of neurotransmission (e.g., dopaminergic, GABAergic, serotoninergic) by different axon guidance molecules remain unknown. However, it is known that axon guidance molecules are involved in glutamatergic transmission and long term potentiation (60). Therefore, oxycodone induced alterations in axon guidance gene expression may be relevant to neuronal plasticity which may occur in addictive-like state (1, 62). This is the first RNAseq study on the impact of a chronic period of oxycodone self-administration, a widely abused prescription drug on expression of axon guidance genes in the mouse brain.

In conclusion, we have found alterations in expression of specific axon guidance genes in the mouse striatum following chronic oxycodone self-administration. Although, their exact functions in drug taking or drug seeking behaviors are not known, these proteins are promising targets for further studies and development of treatment of oxycodone addiction.

## Author contributions

YZ and MK designed the experiment. YZ and CZ performed experiments. YL, MR, and VY analyzed data, and drafted the manuscript.

### Conflict of interest statement

The authors declare that the research was conducted in the absence of any commercial or financial relationships that could be construed as a potential conflict of interest.
